# Chlorotoxin peptide-functionalized polyethylenimine-entrapped gold nanoparticles for glioma SPECT/CT imaging and radionuclide therapy

**DOI:** 10.1186/s12951-019-0462-6

**Published:** 2019-02-19

**Authors:** Lingzhou Zhao, Yujie Li, Jingyi Zhu, Na Sun, Ningning Song, Yan Xing, He Huang, Jinhua Zhao

**Affiliations:** 1 Department of Nuclear Medicine, Shanghai General Hospital, Shanghai Jiao Tong University School of Medicine, Shanghai, 200080 People’s Republic of China; 2 State Key Laboratory of Material-Oriented Chemical Engineering, School of Pharmaceutical Sciences, Nanjing Tech University, Nanjing, 211816 People’s Republic of China

**Keywords:** Polyethyleneimine, Chlorotoxin, Glioma, Gold nanoparticles, SPECT/CT imaging, Radionuclide therapy

## Abstract

**Background:**

Malignant glioma is the most common and deadliest brain cancer due to the obstacle from indistinct tumor margins for surgical excision and blood brain barrier (BBB) for chemotherapy. Here, we designed and prepared multifunctional polyethylenimine-entrapped gold nanoparticles (Au PENPs) for targeted SPECT/CT imaging and radionuclide therapy of glioma.

**Results:**

Polyethylenimine was selected as a template for sequential modification with polyethylene glycol (PEG), glioma-specific peptide (chlorotoxin, CTX) and 3-(4-hydroxyphenyl)propionic acid-OSu (HPAO), and were then used to entrap gold nanoparticles (Au NPs). After ^131^I radiolabeling via HPAO, the ^131^I-labeded CTX-functionalized Au PENPs as a multifunctional glioma-targeting nanoprobe were generated. Before ^131^I radiolabeling, the CTX-functionalized Au PENPs exhibited a uniform size distribution, favorable X-ray attenuation property, desired water solubility, and cytocompatibility in the given Au concentration range. The ^131^I-labeled CTX-functionalized Au PENPs showed high radiochemical purity and stability, and could be used as a nanoprobe for the targeted SPECT/CT imaging and radionuclide therapy of glioma cells in vitro and in vivo in a subcutaneous tumor model. Owing to the unique biological properties of CTX, the developed nanoprobe was able to cross the BBB and specifically target glioma cells in a rat intracranial glioma model.

**Conclusions:**

Our results indicated that the formed nanosystem had the significant potential to be applied for glioma targeted diagnosis and therapy.

**Electronic supplementary material:**

The online version of this article (10.1186/s12951-019-0462-6) contains supplementary material, which is available to authorized users.

## Introduction

Malignant glioma is the most aggressive and lethal form of brain cancer [1, 2]. Patients with gliomas suffer from a poor prognosis, and the 5-year survival rate of high grade gliomas is less than 10% [3–5]. Owing to the special pathological features of glioma cells, the tumor margin in the brain is often indistinct, and complete tumor resection is rarely achieved through surgery. The blood brain barrier (BBB) is the main obstacle to chemotherapy treatment and its physiological characteristics limit the available treatment options for gliomas, often leading to unsatisfactory clinical outcomes [6]. The precise boundary definition and treatment of gliomas therefore remains challenging.

To meet this challenge, nanoparticle-based platforms have been widely investigated owing to their potential clinical values for the diagnosis and treatment of diseases [7–14]. Nanoplatforms are capable of precise diagnosis, drug delivery, and therapy monitoring through integrating diagnostic and therapeutic functions [15–18]. For example, the development of various radionuclide labeled nanoparticles combined with other imaging modalities or treatment techniques, could make them appropriate for a wider variety of applications [19–21]. To date, various methods have been adopted for engineering BBB-penetrating nanoparticle-based theranostic systems for glioma imaging and therapy [22–26]. Among these methods, attaching ligands, such as specific antibodies or peptides, to nanoparticles, has been reported to be one of the most promising strategies [26].

Chlorotoxin (CTX), a 36-amino acid peptide, has been shown to be a tumor-targeting ligand owing to its strong affinity for a range of tumors, including glioma, medulloblastoma, prostate cancer, sarcoma, and intestinal cancer [27–29]. In addition, CTX has been observed to permeate intact BBBs in both animal models and humans with brain tumors [29]. Owing to the unusual specificity of CTX for glioma and its ability to penetrate the BBB, radionuclide ^131^I and indocyanine green (ICG) labeled CTX have been used for targeted imaging and treatment of glioma in clinical trials [29–31]. Meanwhile, many types of CTX-conjugated nanoparticles have emerged in this field, which not only have the capability to cross the BBB and selectively bind to glioma cells, but are also endowed with imaging and therapeutic functionalities [32–35]. For instance, an early study showed that CTX-conjugated magnetic nanoparticles could cross the BBB and be used for magnetic resonance (MR)/fluorescence imaging of glioma, whereas those without CTX conjugation were hindered [27]. It has also been shown that CTX-modified liposomes and polyethylenimine (PEI) could be developed as drug and gene delivery systems for glioma-targeted chemotherapy and gene therapy [35–37]. In our recent work, we developed CTX-conjugated multifunctional dendrimers labeled with radionuclides for targeted imaging and therapy of glioma [38]. Furthermore, we have demonstrated that radionuclide-labeled dendrimers can be used to entrap gold nanoparticles (Au NPs) for tumor dual mode imaging applications, such SPECT/CT and SPECT/MR imaging [39–41]. The previous successes of dendrimer-based multifunctional nanosystems and the unique biological properties of CTX, prompted our investigation of dendrimers as a BBB-penetrating nanoplatform for the dual mode imaging and radionuclide therapy of glioma.

Hence, in this study, PEI dendrimers were sequentially conjugated with polyethylene glycol (PEG), CTX, and 3-(4-hydroxyphenyl)propionic acid-OSu (HPAO) for facile ^131^I radiolabeling, and then the functionalized PEI was used as a template to entrap Au NPs. Followed by acetylation of the remaining terminal amines and radioiodination, the designed {(Au^0^)_200_-PEI.NHAc-FI-^131^I-HPAO-(PEG-CTX)-*m*PEG} NPs (^131^I-Au PENPs-CTX) as a theranostic nanoplatform were prepared. The formed CTX-modified Au PENPs were well characterized before and after ^131^I radiolabeling using different techniques to determine the structure, size, X-ray attenuation property, cytotoxicity, stability, and specific targeting to glioma cells in vitro. The prepared ^131^I-Au PENPs-CTX were then used for targeted SPECT/CT imaging and radionuclide therapy of glioma cells in vitro and in vivo using a subcutaneous glioma tumor model. Finally, an intracranial rat model was used to investigate the penetration of ^131^I-Au PENPs-CTX across the BBB and the specific recognition of glioma cells in the brain.

## Experimental section

### Materials

PEI (Branched, Mw = 25,000), chloramine-T trihydrate (ch-T), HPAO, 1-butanethiol, potassium iodide (KI), acetic anhydride (Ac_2_O), triethylamine (TEA), phosphate buffered saline (PBS), and 1-ethyl-3-(3-(dimethylamino)propyl) carbodiimide hydrochloride (EDC) were supplied by Sigma-Aldrich (St. Louis, MO). PEG monomethyl ether with one end terminating in a carboxyl group (*m*PEG-COOH, Mw = 5000), PEG with one end terminating in a maleimide group and the other in a succinimidyl valerate group (MAL-PEG-SVA, Mw = 5000), and cellulose dialysis membranes were obtained from Shanghai Yanyi Biotechnology Corporation (Shanghai, China). CTX peptide was manufactured by Shanghai Bootech BioScience & Technology Co., Ltd. (Shanghai, China). Na^131^I solution was obtained from Shanghai GMS Pharmaceutical Co., Ltd. (Shanghai, China). Disposable PD-10 desalting columns, fluorescein isothiocyanate (FI), cell counting kit-8 (CCK-8), C6 glioma cells, fetal bovine serum (FBS), RPMI 1640 medium, penicillin, and streptomycin were procured from Shanghai Dobio CO., Ltd. (Shanghai, China). HAuCl_4_·4H_2_O, sodium borohydride (NaBH_4_), and all other chemicals and solvents were supplied by Sinopharm Chemical Reagent Co., Ltd. (Shanghai, China).

### Synthesis of {(Au^0^)_200_-PEI.NHAc-FI-^131^I-HPAO-(PEG-CTX)-*m*PEG} NPs

First, CTX-conjugated PEI-NH_2_ was prepared according to our previously reported method [38]. In brief, *m*PEG-COOH (300 mg) per-activated by EDC (175.2 mg), and PEI.NH_2_ (100.0 mg) dissolved in DMSO were mixed under constant stirring for 3 days to obtain the PEI.NH_2_-*m*PEG. MAL-PEG-SVA (300.0 mg) dissolved in DMSO was then added dropwise to the mixture over another 3 days, and CTX peptide (114.8 mg) was reacted with the MAL groups on the PEI surface overnight to synthesize the PEI.NH_2_-(PEG-CTX)-*m*PEG conjugates. The remaining MAL groups were then blocked using excess 1-butanethiol to avoid the reaction with HPAO in the following step. HPAO (31.6 mg) and FI (7.8 mg) were then sequentially added to the reaction mixture with stirring overnight to obtain the PEI.NH_2_-HPAO-FI-(PEG-CTX)-*m*PEG.

Second, PEI-entrapped Au NPs were prepared using NaBH_4_ reduction chemistry following the protocols reported in our previous work [38]. Briefly, HAuCl_4_ solution (0.01 M, 80 mL) was reacted with the PEI.NH_2_-HPAO-FI-(PEG-CTX)-*m*PEG (768.5 mg, 20 mL) at a molar ratio 200:1 with stirring for 0.5 h. After the addition of cold NaBH_4_ solution (10.0 mg/mL, 9.1 mL) over 2 h, the crude {(Au^0^)_200_-PEI.NH_2_-HPAO-FI-(PEG-CTX)-*m*PEG} NPs were formed, which were further acetylated by mixing with TEA (1957.1 μL) and Ac_2_O (1107.6 μL) with stirring for 24 h. After purification using a 14 kDa molecular weight cut-off membrane against PBS (3 times, 2 L) and water (5 times, 2 L) over 3 days to remove the excess reactants and byproducts, the final {(Au^0^)_200_-PEI.NHAc-HPAO-FI-(PEG-CTX)-*m*PEG} NPs (Au PENPs-CTX) were acquired. For comparison, Au PENPs without CTX were also synthesized using the same method. The intermediates were collected and characterized to assess the average number of conjugated molecules (HPAO, CTX, *m*PEG, and FI) per PEI dendrimer.

Finally, the Au PENPs-CTX were labeled with ^131^I via the HPAO ligands. Briefly, Na^131^I solution (20 mCi, 200 μL) was mixed with chloramine T (200 μg) and Au PENPs-CTX (200 μg) dissolved in 250 μL of PBS (0.1 M, pH = 7.2–7.4) with continuous stirring. After incubation for 30 min at 37 °C, the reaction mixture was purified using PD-10 desalting columns with saline as the mobile phase. The ^131^I-Au PENPs-CTX were collected and ^131^I-Au PENPs without CTX modification were also prepared using the same experimental conditions. Their radiostabilities in vitro were assessed by measuring the radiochemical purities at different time intervals using instant thin-layer chromatography (ITLC).

### In vitro SPECT and CT imaging

In vitro SPECT and CT imaging were used to confirm the targeting specificity of CTX-functionalized Au PENPs for glioma cells. In brief, C6 cells were seeded in 12-well plates at a density of 2 × 10^5^ cells/well. After incubation for 24 h, the culture medium was replaced with 2 mL of fresh medium containing ^131^I-Au PENPs-CTX or ^131^I-Au PENPs at different radioactivity concentrations (25, 50, 100, 200, and 400 μCi/mL), and incubation was continued for an additional 4 h. The cells were then harvested and washed 3 times with PBS, centrifuged in 1.5 mL tubes, and in vitro SPECT imaging was performed. Similarly, after incubation with Au PENPs-CTX and Au PENPs at different Au concentrations (12.5, 25, 50, and 100 μM, respectively) for 4 h and collecting the treated cells via the same procedure, in vitro CT imaging was also studied.

### Targeted SPECT/CT imaging and radionuclide therapy of glioma in a subcutaneous glioma tumor model

Animal experiments were performed using protocols approved by the ethical committee of Shanghai General Hospital. BALB/c female nude mice (20–22 g) were purchased from Shanghai Slac Laboratory Animal Center (Shanghai, China) and subcutaneously injected with 2 × 10^6^ C6 cells in the right side flank. When the tumor volume reached approximately 0.8 cm^3^, the mice were given water containing 1% KI for 3 days to saturate the thyroid.

Before SPECT imaging in vivo, the mice were divided into targeted and non-targeted groups (n = 5 per group) at random. The mice in the targeted group were intravenously administrated a PBS solution of ^131^I-Au PENPs-CTX (400 μCi, 100 μL), and those in the non-targeted group were injected with ^131^I-Au PENPs at the same dose for comparison. SPECT images were then captured at 0.5, 2, 4, 6, 8, and 16 h post-injection. At 8 h post-injection, one mouse from each group was euthanized, and the tumors and major organs (heart, lung, liver, stomach, spleen, kidneys, soft tissue, and intestines) were collected for analysis of the relative signal intensities. For CT imaging in vivo, the mice in the targeted and non-targeted groups were intravenously injected with Au PENPs-CTX and Au PENPs ([Au] = 0.1 M, 100 μL), respectively, and scanned before dosing and at different time points post-injection (0.5, 2, 4, 6, 8, and 16 h) using the CT system.

We next evaluated the therapeutic efficacy of ^131^I-Au PENPs-CTX in vivo in a subcutaneous tumor model. Two weeks after tumor inoculation, the mice were split randomly into 5 groups (5 mice per group). The mice in each group were sequentially treated with ^131^I-Au PENPs-CTX, ^131^I-Au PENPs, Au PENPs-CTX, Au PENPs, or saline via intravenous injection at an Au concentration of 0.1 M in 100 μL PBS solution (with 200 μCi per mouse of ^131^I in the ^131^I-Au PENPs-CTX and ^131^I-Au PENPs groups). The treatments were then given every 3 days, a total of 7 times, and the tumor size and body weight of each mouse were measured after each injection. The relative tumor volumes, body weight, and survival rate were calculated as described in our previous work [38].

### Histological examinations

To investigate the potential toxicity of the ^131^I-Au PENPs-CTX in vivo, one mouse from each group treated with ^131^I-Au PENPs-CTX, ^131^I-Au PENPs, Au PENPs-CTX, Au PENPs, or saline was euthanized to extract the tumor and major organs (heart, liver, spleen, lung, and kidneys) after 21 days of treatment. The excised tumors and organs were fixed, embedded, sectioned, and stained with hematoxylin and eosin (H&E), and then observed using an AMEX 1200 inverted phase contrast microscope (Thermo Fisher Scientific, Inc.). Furthermore, tumor apoptosis was analyzed using a terminal deoxynucleotidyl transferase dUTP nick end labeling (TUNEL) method and an apoptotic detection kit (Roche, Basel, Switzerland). The TUNEL kit was used for fixation, dehydration, paraffin-embedding, sectioning, and staining; then the tumor sections were observed with a Leica DM IL LED inverted phase contrast microscope.

### Targeted SPECT imaging of glioma in an orthotopic glioma rat model

To demonstrate the ability of CTX-modified Au PENPs to penetrate the BBB and target glioma cells, orthotopic glioma rat models were prepared. The male SD rats (150–180 g) were anesthetized with 5% chloral hydrate and individually placed in a stereotaxic apparatus. Subsequently, 1 × 10^7^ C6 cells suspended in 10 μL of sterile PBS solution were injected into the right brain of each rat (striatum, 1 mm lateral to bregma and 3 mm deep from the dura) at a rate of 1 μL/min. Two weeks after the injection, the rats were given water containing 1% KI for 3 days to block the thyroid uptake of ^131^I, and randomly divided into the experimental and control groups (5 rats per group). ^131^I-Au PENPs-CTX (800 μCi, 200 μL) were then injected into the rat in the experimental group via the tail vein. For comparison, ^131^I-Au PENPs without CTX were also administered to the control group at the same dose. SPECT images were acquired at 0.5, 2, 4, 6, 8, and 16 h post-injection, and one rat from each group was euthanized at 16 h post-injection to measure the relative radioactivity intensities in the brain.

### Statistical analysis

The significance of the experimental data was assessed by one-way ANOVA. A p value of 0.05 was considered significant, and data are denoted (**) for p < 0.01 and (***) for p < 0.001.

## Results and discussion

### Synthesis and characterization of {(Au^0^)_200_-PEI.NHAc-FI-^131^I-HPAO-(PEG-CTX)-*m*PEG} NPs

**Fig. 1:**
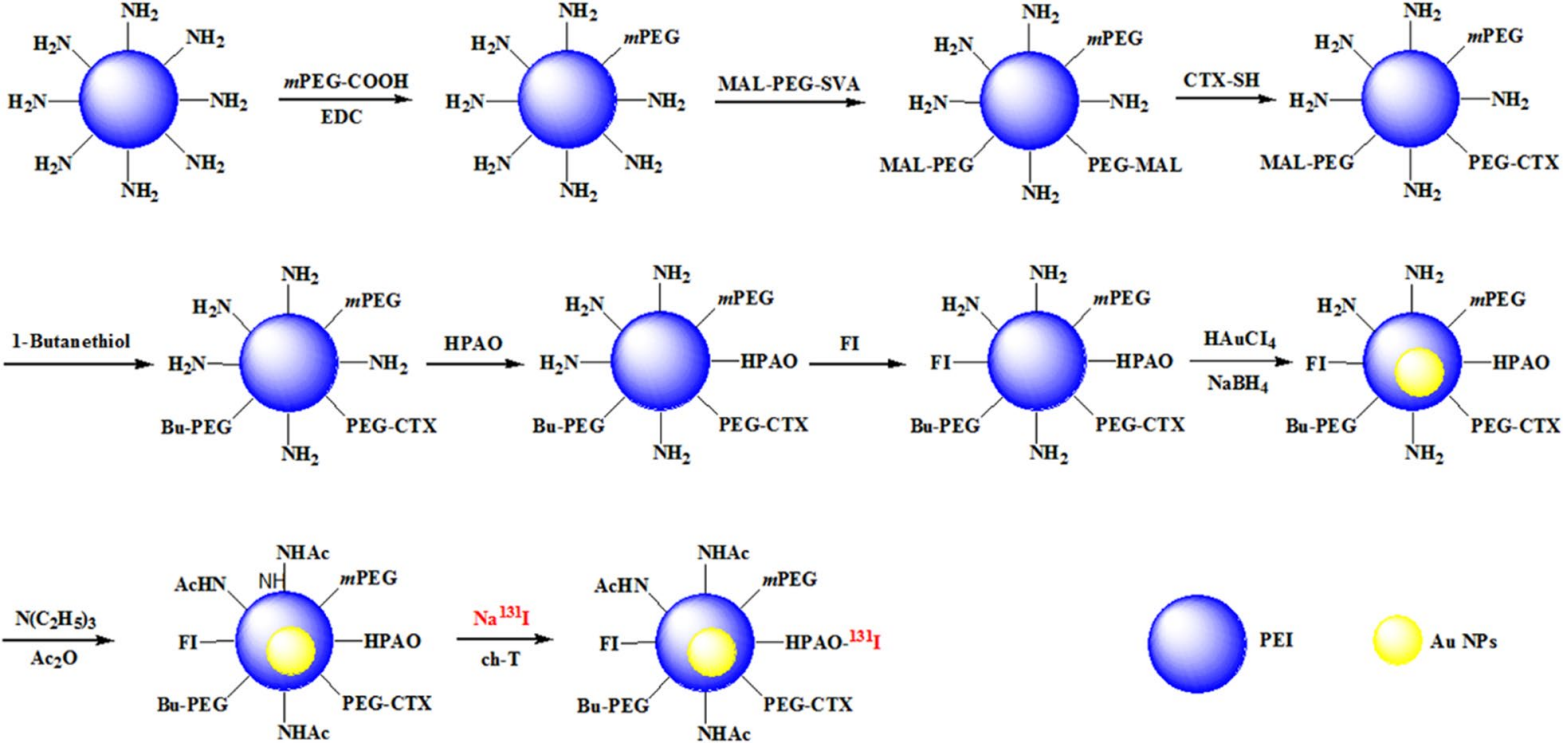
Schematic illustration of the synthesis of the ^131^I-Au PENPs-CTX

In this study, PEI dendrimers were sequentially modified with *m*PEG-COOH, CTX via a PEG linker, HPAO, and FI to give a multifunctional PEI-based template for the entrapment of Au NPs. Followed by acetylation of the remaining PEI terminal amines and radiolabeling with ^131^I, the ^131^I-Au PENPs-CTX were manufactured (Fig. 1).

^1^H NMR spectroscopy was used to characterize the PEI-based templates of PEI.NH_2_-HPAO-FI-(PEG-CTX)-*m*PEG and PEI.NH_2_-HPAO-FI-(PEG-MAL)-*m*PEG, and intermediate products including PEI.NH_2_-*m*PEG, PEI.NH_2_-(PEG-MAL)-*m*PEG, PEI.NH_2_-(PEG-CTX)-*m*PEG, and PEI.NH_2_-HPAO-(PEG-CTX)-*m*PEG. The average number of *m*PEG, PEG-MAL, CTX, HPAO, and FI units conjugated to each PEI was calculated based on NMR integration and is recorded in Additional file 1: Table S1 (ESI). The characterization of these conjugates is shown in Additional file 1: Fig. S1a–g (ESI).

ICP-OES was performed to confirm the Au content of the PEI-entrapped Au NPs. The Au salt/PEI molar ratio selected was 200:1 and the ICP results suggested that the Au(III) salt was completely reduced to Au(0) in the Au PENPs-CTX and Au PENPs, with average numbers of Au atoms per PEI close to the selected Au salt/PEI molar ratio. Moreover, the successful formation of Au NPs within PEI was demonstrated using UV–vis spectroscopy. As shown in Additional file 1: Fig. S1h, an apparent absorption feature is present at 540 nm due to the particle-induced light scattering effect and the surface plasmon resonance peak of Au NPs, in agreement with the results reported in the literature [39, 41]. The stabilities of the Au PENPs-CTX and Au PENPs were further assessed using UV–vis spectroscopy under different temperature and pH conditions (Additional file 1: Fig. S2a, b). The results show that the absorption features of Au PENPs-CTX and Au PENPs exhibit negligible changes, suggesting good colloidal stability in the given pH (5.0–8.0) and temperature (4–50 °C) ranges.

**Fig. 2:**
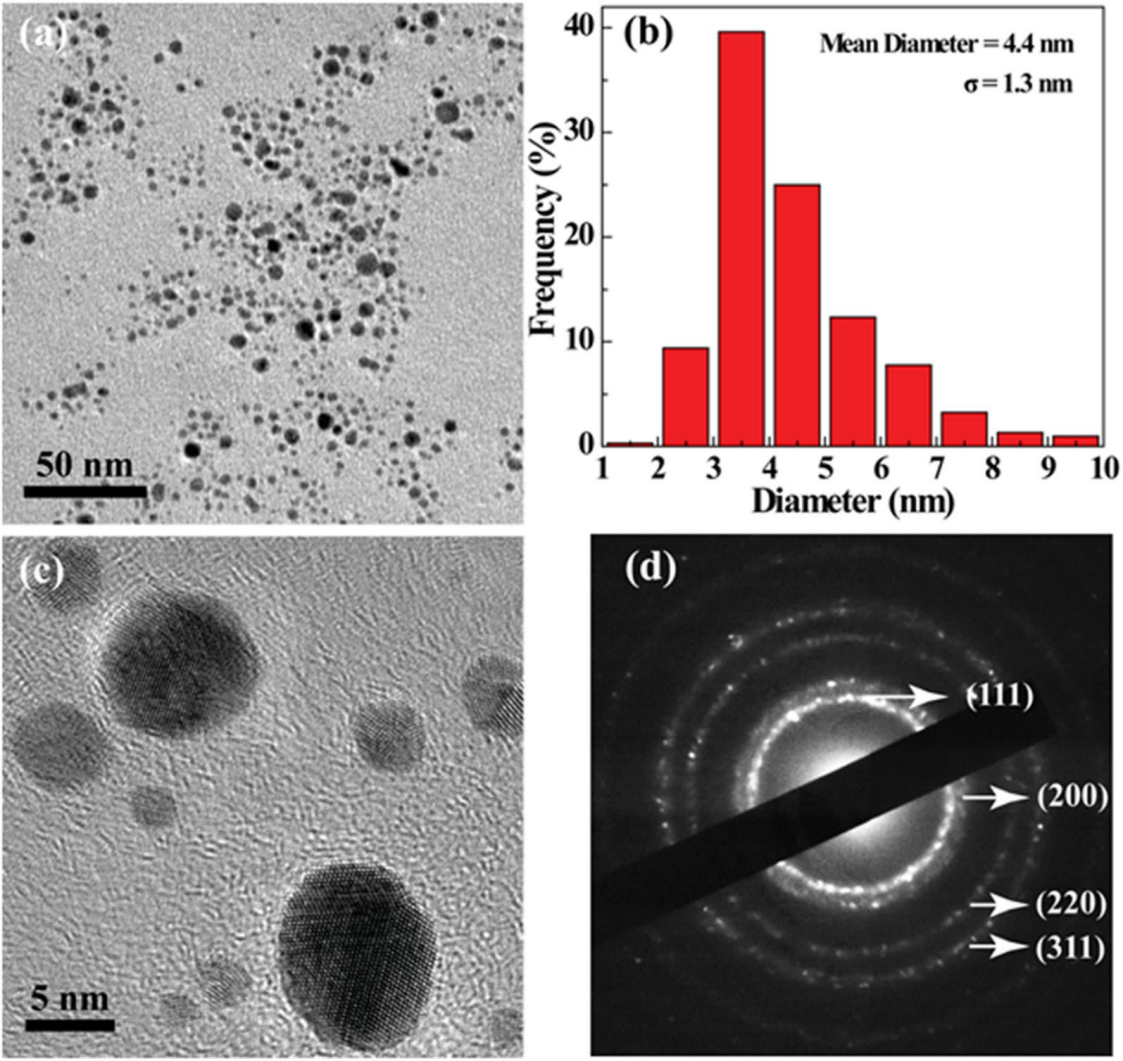
**a** TEM image, **b** size distribution histogram, **c** high-resolution TEM image, and **d** selected area electron diffraction pattern of Au PENPs-CTX

Dynamic light scattering (DLS) was carried out to measure the average hydrodynamic sizes of Au NPs, and the data suggested that the Au PENPs-CTX and Au PENPs had a hydrodynamic diameter of 151.0 ± 25.4 and 147.1 ± 9.0 nm with a low polydispersity index (0.47 ± 0.05 and 0.31 ± 0.06), respectively. Furthermore, the hydrodynamic sizes of Au PENPs-CTX and Au PENPs had little appreciable changes after being stored in DMEM supplemented with 10% FBS for a period of 48 h, confirming the favorable colloidal stability. The morphology and size of Au PENPs-CTX were also analyzed by TEM (Fig. 2). The results revealed that Au PENPs-CTX had a spherical shape with relatively narrow size distribution. Both high-resolution TEM image and selected area electron diffraction (SAED) pattern showed high crystallinity of the Au core NPs, and the featured (111), (200), (220), and (311) rings could be used to prove the face-centered-cubic crystal structure of the formed Au NPs. The Au core size of the Au PENPs-CTX observed by TEM was 4.4 ± 1.3 nm, notably smaller than the hydrodynamic size obtained from DLS. This may be owing to the fact that TEM shows the Au core of a single particle, where DLS measures the aggregation of numerous single Au PENPs in water. The zeta potentials of the Au PENPs-CTX and Au PENPs were calculated to be 5.09 ± 0.05 and 11.67 ± 0.23 mV, respectively, which was near neutral and further supported the successful acetylation of the remaining surface amine groups of PEI-entrapped Au NPs by Ac_2_O.

Followed by ^131^I radiolabeling with HPAO onto the surface of Au PENPs-CTX, the multifunctional nanoprobe for targeted SPECT/CT imaging and radionuclide therapy of glioma was developed. ITLC was used to assess the radiochemical yield and radiostability of the ^131^I-labeled PEI-entrapped Au NPs (Additional file 1: Fig. S2c, d). The findings indicated that the radiolabeling efficiency of ^131^I-Au PENPs-CTX and ^131^I-Au PENPs was 60.4 ± 5.4 and 64.5 ± 6.8% (n = 3), respectively. After PD-10 column purification, the radiochemical purities of both ^131^I-Au PENPs-CTX and ^131^I-Au PENPs were over 99%, and they remained above 90% after 24 h in PBS at room temperature and in FBS at 37 °C, indicating excellent radiostabilities.

### In vitro cytotoxicity

The potential cytotoxicity of the CTX-modified Au PENPs before and after ^131^I radiolabeling was assessed using a cell counting kit-8 (CCK-8) assay in vitro (Additional file 1: Fig. S3). It was clear that the C6 cells incubated with Au PENPs-CTX and Au PENPs without ^131^I radiolabeling had high viabilities even at Au concentrations up to 200 μM, and no appreciable difference was observed when compared with the control cells treated with PBS (P > 0.05), suggesting excellent cytocompatibility. In contrast, C6 cells were slightly inhibited in a dose dependent manner after treatment with ^131^I-Au PENPs for 24 h, and the cell viability was less than 80% at the radioactivity concentration of 200 μCi/mL. In addition, a more obvious inhibition trend was found for the C6 cells incubated with ^131^I-Au PENPs-CTX, which showed inhibition rates up to 59% at a radioactivity concentration of 200 μCi/mL, as a result of the enhanced cellular uptake and ^131^I delivery of CTX-modified Au PENPs.

### Specific targeting of {(Au^0^)_200_-PEI.NHAc-FI-^131^I-HPAO-(PEG-CTX)-*m*PEG} NPs to glioma cells

CTX has been shown to have targeting specificity for matrix metalloproteinase 2 (MMP2) on the surface of glioma cells. To investigate the affinity of CTX-modified Au PENPs for C6 cells, the cellular uptake was analyzed by flow cytometry (Additional file 1: Fig. S4) and confocal microscopy (Additional file 1: Fig. S5). As shown in Additional file 1: Fig. S4, significantly higher fluorescence intensities are measured for C6 cells treated with Au PENPs-CTX (5 μM), while lower fluorescence is observed for cells treated with Au PENPs at the same concentration. These data confirmed that CTX-factionalized Au PENPs were able to specifically target glioma cells presumably via specific MMP2 interaction. The targeting specificity of the Au PENPs-CTX for C6 cells was also demonstrated by confocal microscopy. After a 2 h incubation with Au PENPs-CTX (5.0 μM), C6 cells displayed much stronger fluorescence intensities than those treated with Au PENPs or PBS under the same conditions. Taken together, the results of the flow cytometry assay and confocal microscope imaging, led to the conclusion that the CTX peptide modification might promote the cellular uptake of Au PENPs via a receptor-mediated pathway.

### In vitro CT and SPECT imaging of C6 cells

**Fig. 3:**
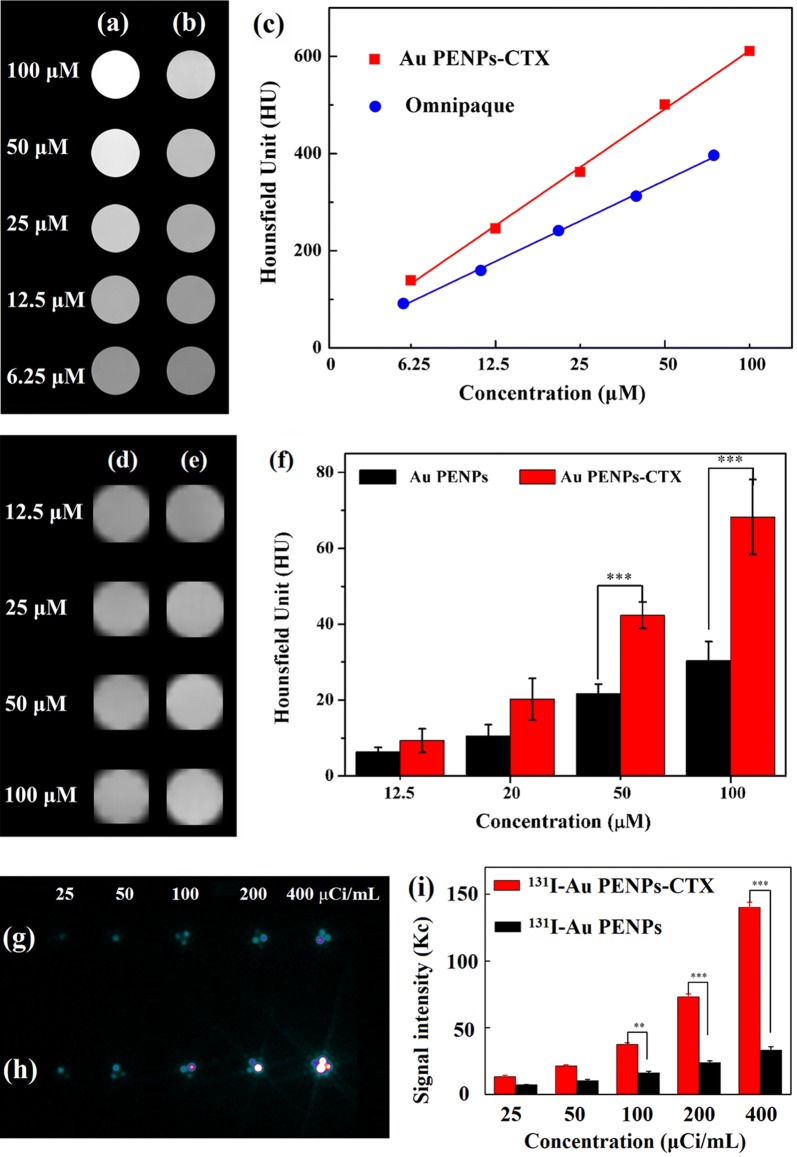
CT images of **a** Au PENPs-CTX and **b** Omnipaque at different concentrations of Au or I, and **c** their X-ray attenuation intensities. In vitro CT images of C6 cells treated with **d** Au PENPs and **e** Au PENPs-CTX for 4 h at the Au concentrations of 12.5, 25, 50 and 100 μM, respectively, and **f** their quantitative CT values. In vitro SPECT images of C6 cells treated with **g** ^131^I-Au PENPs and **h** ^131^I-Au PENPs-CTX for 4 h at the radioactive concentrations of 25, 50, 100, 200 and 400 μCi/mL, respectively, and **i** their relative SPECT signal intensities

Before assessing the applicability of ^131^I-Au PENPs-CTX for targeted SPECT/CT imaging of glioma models in mice, the imaging performance was first confirmed in vitro. For CT imaging, the X-ray attenuation properties of the developed Au PENPs-CTX and Omnipaque, a small molecule CT contrast agent used in clinic, were compared. As shown in Fig. 3a, b, the CT images are brighter and their CT values become larger with the increasing concentrations of Au or I. The Au PENPs-CTX possesses much higher HU values than Omnipaque at the same Au or I concentrations in Fig. 3c, resulting in the sharper increase of the Au PENPs-CTX. The results indicated that the X-ray attenuation intensity was enhanced in the same way and the PEI-based Au NPs had a stronger X-ray attenuation property than Omnipaque.

The feasibility of Au PENPs-CTX for C6 cell CT imaging in vitro was studied. Although the brightness of the CT images in Fig. 3d, e appears to show little significant difference between the C6 cells treated with Au PENPs-CTX and those treated with Au PENPs, the quantitative analysis in Fig. 3f shows that C6 cells treated with the CTX-modified Au PENPs have a higher CT value than those treated with the Au PENPs without CTX modification at different Au concentrations ([Au] = 12.5, 25, 50, and 100 μM, respectively). At the Au concentration of 100 μM, the CT value measured for the cells treated with Au PENPs-CTX was 2.2 times higher than that of cells treated with Au PENPs, suggesting that the modification of CTX rendered Au PENPs with targeting specificity to glioma cells.

Finally, the potential of Au PENPs-CTX for targeted SPECT imaging of glioma cells in vitro was validated after ^131^I radiolabeling. As shown in Fig. 3g, h, the SPECT images are brighter for samples with higher ^131^I concentration, and the brightness of the cells treated with ^131^I-Au PENPs-CTX is much stronger than that of the cells treated with ^131^I-Au PENPs, which is verified by quantitative analysis of their relative SPECT signals in Fig. 3i. At the concentration of 400 μCi/mL, the SPECT signal intensity of C6 cells treated with the ^131^I-Au PENPs-CTX was 4.2 times higher than that of cells treated with the ^131^I-Au PENPs. These findings supported the application of ^131^I-labeled CTX-modified Au PENPs for the targeted SPECT imaging of glioma cells in vitro.

### Targeted SPECT/CT imaging and radionuclide therapy for a subcutaneous glioma tumor model in vivo

**Fig. 4:**
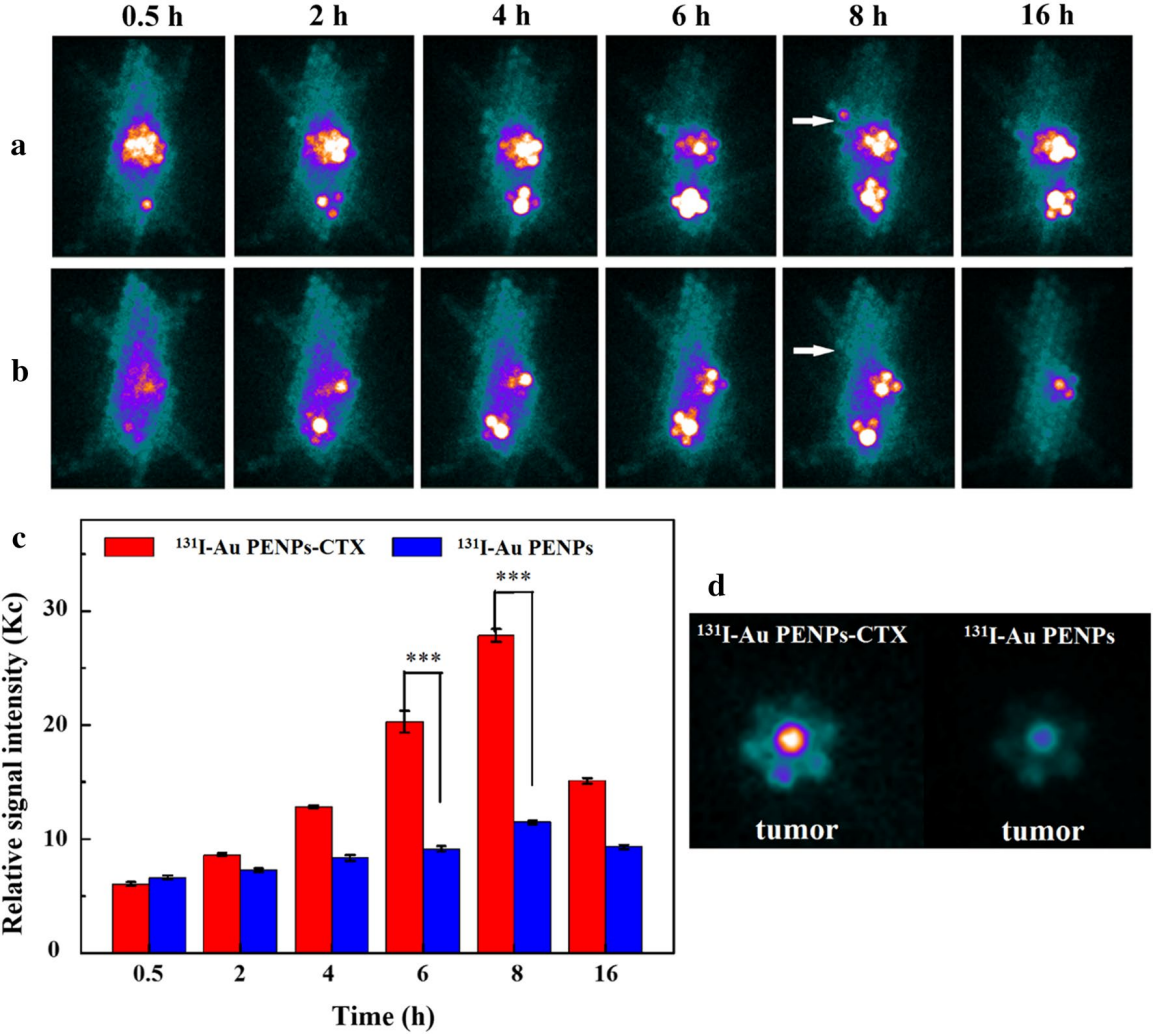
In vivo SPECT images of the nude mice bearing C6 xenografted tumors treated with **a** ^131^I-Au PENPs-CTX and **b** ^131^I-Au PENPs at different time points of 0.5, 2, 4, 6, 8 and 16 h, respectively, and **c** their tumor relative signal intensities. **d** SPECT images of ex vivo tumors at 8 h postinjection. The white arrow points to the tumor site

Based on the successful SPECT and CT imaging of C6 cells in vitro, we investigated the potential of ^131^I-Au PENPs-CTX for the dual mode imaging of a subcutaneous glioma tumor model in vivo. As shown in Fig. 4a, no distinct accumulation of radioactivity can be observed 0.5 h post-injection in the tumors of mice intravenously administrated ^131^I-Au PENPs-CTX. A slightly enhanced tumor uptake is found at 2 h post-injection, followed by much stronger SPECT signal intensities at 4 and 6 h post-injection. The tumor SPECT signal intensity reaches a peak at 8 h post-injection, and can still be detected at 16 h post-injection. In contrast, no visible tumor uptake in Fig. 4b is observed in the mice treated with the ^131^I-Au PENPs at 0.5, 2, 4, 6, or 16 h post-injection, and only a small amount of radioactivity accumulates in the tumor sites at 8 h post-injection as a result of the EPR effect. The SPECT image of ex vivo tumors also shows much higher tumor SPECT signal intensity in the mice treated with ^131^I-Au PENPs-CTX at 8 h post-injection in Fig. 4d. The quantitative SPECT intensity measurements in Fig. 4c show that the mice injected with ^131^I-Au PENPs-CTX have higher tumor signal intensities than those injected with ^131^I-Au PENPs at the same time points. For instance, the tumor signal intensities of the mice injected with ^131^I-Au PENPs-CTX are 2.2 and 2.4 times higher than those of the mice treated with ^131^I-Au PENPs at 6 and 8 h post-injection, respectively. In addition, the biodistribution at 8 h post-injection was determined to confirm the significant difference in the SPECT signal intensity of the tumors. As shown in Additional file 1: Fig. S6, the liver, intestines and stomach are the major organs to assimilate the ^131^I-labeled PEI-entrapped Au NPs, resulting in relatively low radioactive accumulation in the other organs, including the heart, lung, tumor, kidneys, spleen and soft tissue. It should be noted that the tumor uptake of ^131^I-Au PENPs-CTX is much higher than that of ^131^I-Au PENPs, further confirming the targeting function of the CTX peptide conjugated to the PEI.

**Fig. 5:**
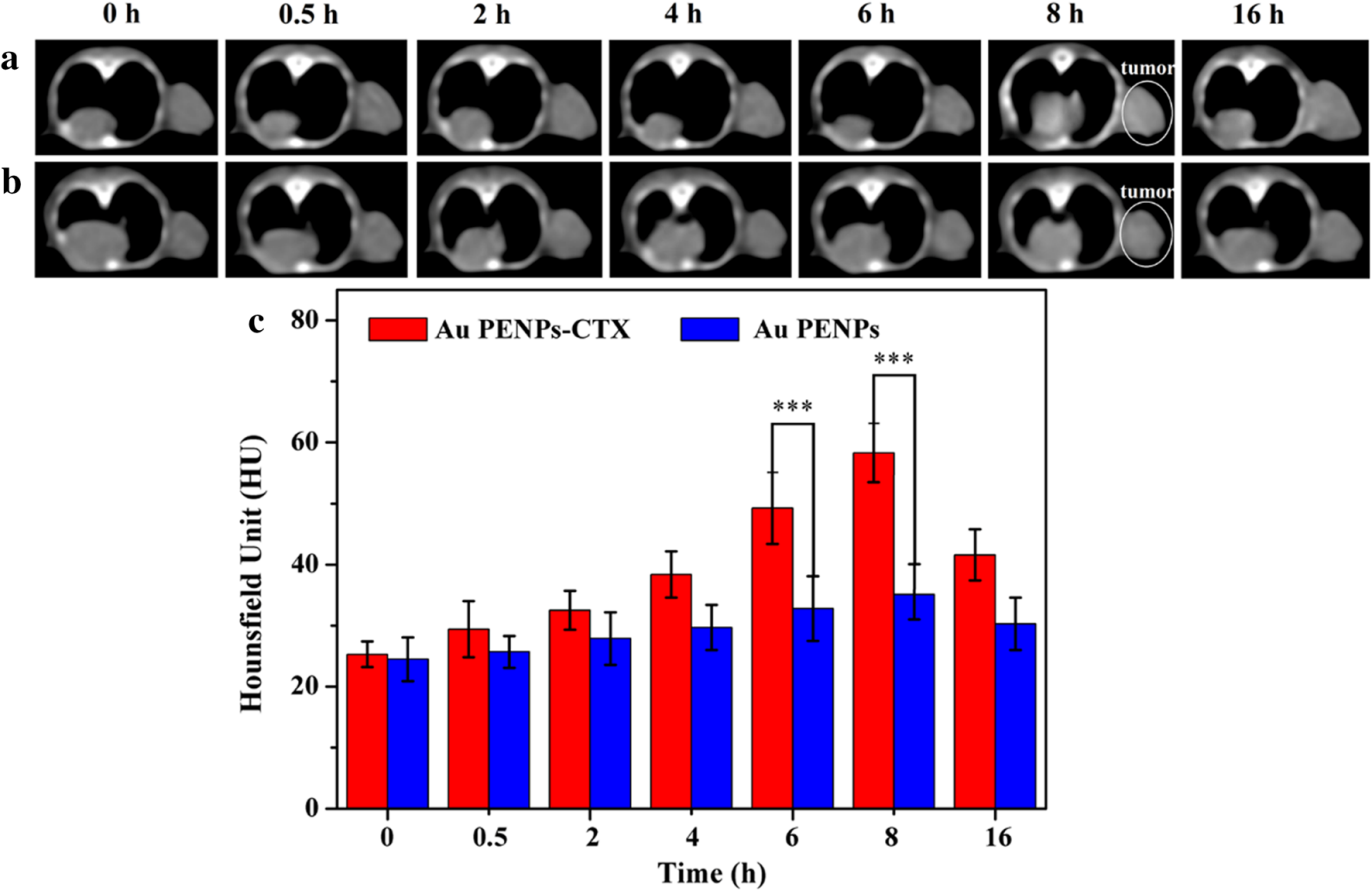
In vivo CT images of the nude mice bearing C6 xenografted tumors treated with **a** Au PENPs-CTX and **b** Au PENPs at different time points of 0, 0.5, 2, 4, 6, 8 and 16 h, respectively, and **c** their tumor CT values. The white circle points to the tumor site

Similar findings were also established from the CT images, owing to the specific role of CTX. As shown in Fig. 5a, b, the tumor CT enhancement continuously increases in the mice treated with the Au PENPs-CTX, and reaches a peak at 8 h post-injection, while the mice treated with the Au PENPs show no significant difference in tumor CT values during the study period. Quantitative measurements 8 h post-injection in Fig. 5c indicate that the mice treated with the Au PENPs-CTX show a 1.7 times higher tumor CT value than those treated with the Au PENPs. Taken together the results of SPECT and CT, lead to the conclusion that the prepared ^131^I-Au PENPs-CTX have the potential to be used as a nanoprobe for SPECT/CT imaging of glioma in vivo.

**Fig. 6:**
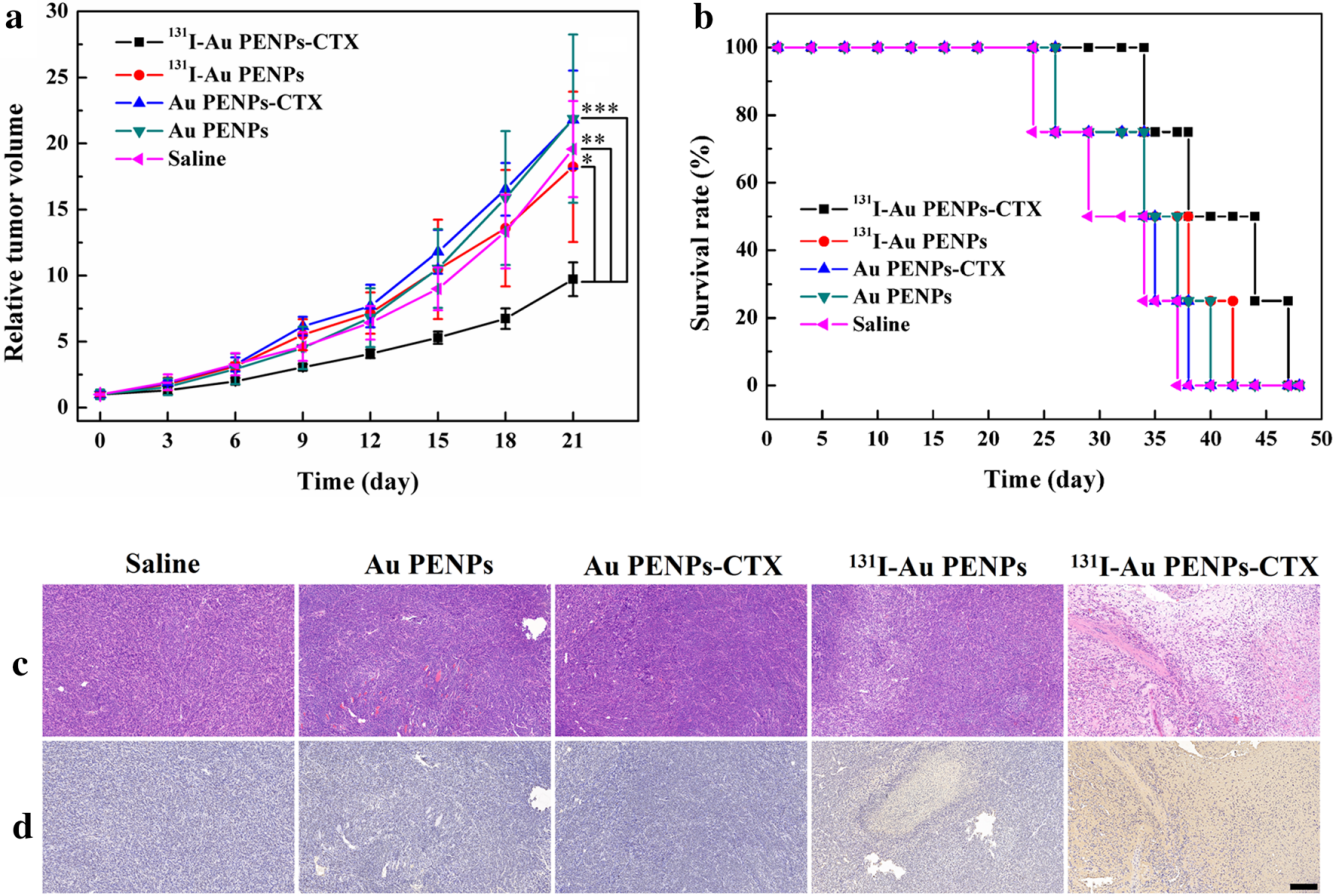
**a** Growth of C6 xenografted tumors in nude mice treated with saline, Au PENPs, Au PENPs-CTX, ^131^I-Au PENPs, and ^131^I-Au PENPs-CTX, respectively. The relative tumor volume was normalized according to their initial tumor volume (mean ± SD, n = 5). **b** Survival rate, **c** H&E staining and **d** TUNEL assay of C6 tumor-bearing mice after various treatments (mean ± SD, n = 5). The scale bar shown in both panels represents 200 μm

The potential to use the ^131^I-Au PENPs-CTX for targeted radionuclide therapy of glioma in vivo was demonstrated. As shown in Fig. 6a, the tumor volume of the mice treated with ^131^I-Au PENPs-CTX increases much more slowly than those of mice treated with PEI-based Au NPs without CTX modification or ^131^I radiolabeling, or the saline control. After 7 treatments, the tumor volumes in each group had increased 9.7 ± 1.3 (^131^I-Au PENPs-CTX), 18.2 ± 5.7 (^131^I-Au PENPs), 21.8 ± 3.7 (Au PENPs-CTX), 21.9 ± 6.4 (Au PENPs) and 19.6 ± 3.6 (saline) times. The tumor inhibition of ^131^I-Au PENPs-CTX is attributed to the enhanced uptake via CTX-mediated targeting to glioma cells, which was also demonstrated by the survival rates of the tumor-bearing mice (Fig. 6b). The survival time of mice treated with ^131^I-Au PENPs-CTX was significantly longer than that of mice in other groups. 25% of the mice survived after treatment with ^131^I-Au PENPs-CTX at 48 days, in comparison the survival time at this percentage was 34 (^131^I-Au PENPs), 32 (Au PENPs-CTX), 34 (Au PENPs), and 33 (saline) days. This further demonstrated that the targeted ^131^I delivery to glioma induced by CTX modification of PEI could produce therapeutic effects with prolonged survival time. Notably, no significant difference in tumor growth was observed among the mice treated with Au PENPs-CTX, Au PENPs, and saline. The reason for this could be that antitumor efficacy results exclusively from the ^131^I radiolabel on PEI. In addition, no significant weight loss was measured in any of the groups of mice (Additional file 1: Fig. S7), suggesting that the ^131^I-Au PENPs-CTX exhibited favorable in vivo safety.

### H&E staining and TUNEL assay

H&E and TUNEL staining were used to further assess the safety and therapeutic effect of the developed ^131^I-Au PENPs-CTX in vivo. H&E staining (Fig. 6c) showed that necrotic regions were only observed in the mice treated with PEI-entrapped Au NPs labeled with ^131^I, and the tumor necrotic area of the mice injected with ^131^I-Au PENPs-CTX was much larger than that of the mice injected with ^131^I-Au PENPs. In contrast, few tumor necrotic cells were observed in the mice treated with Au PENPs-CTX, Au PENPs, and saline. The tumor inhibition efficacy of ^131^I-Au PENPs-CTX was also demonstrated using a TUNEL assay (Fig. 6d). The positive staining of apoptotic cells was only found in the tumors treated with the ^131^I-labeled Au NPs, and the region of apoptotic cells was more evident in the tumors treated with ^131^I-Au PENPs-CTX when compared with ^131^I-Au PENPs. The tumors treated with Au PENPs-CTX, Au PENPs, and saline showed very few positive apoptotic cells in the tumor sections. The H&E and TUNEL staining results indicated that the CTX modification introduced targeting specificity for MMP2-overexpressing tumors into ^131^I-Au PENPs-CTX, for effective radionuclide therapy.

H&E staining was used to further investigate the potential histological toxicity of the formed ^131^I-Au PENPs-CTX and ^131^I-Au PENPs in the major organs, including heart, liver, spleen, lungs, and kidneys (Additional file 1: Fig. S8). The results demonstrated that there was no obvious tissue damage, and no necrotic areas or abnormalities were detected in the organs after 7 treatments, revealing that the developed nanoprobes displayed excellent organ compatibility.

### Targeted SPECT imaging of glioma in an orthotopic rat glioma model

**Fig. 7:**
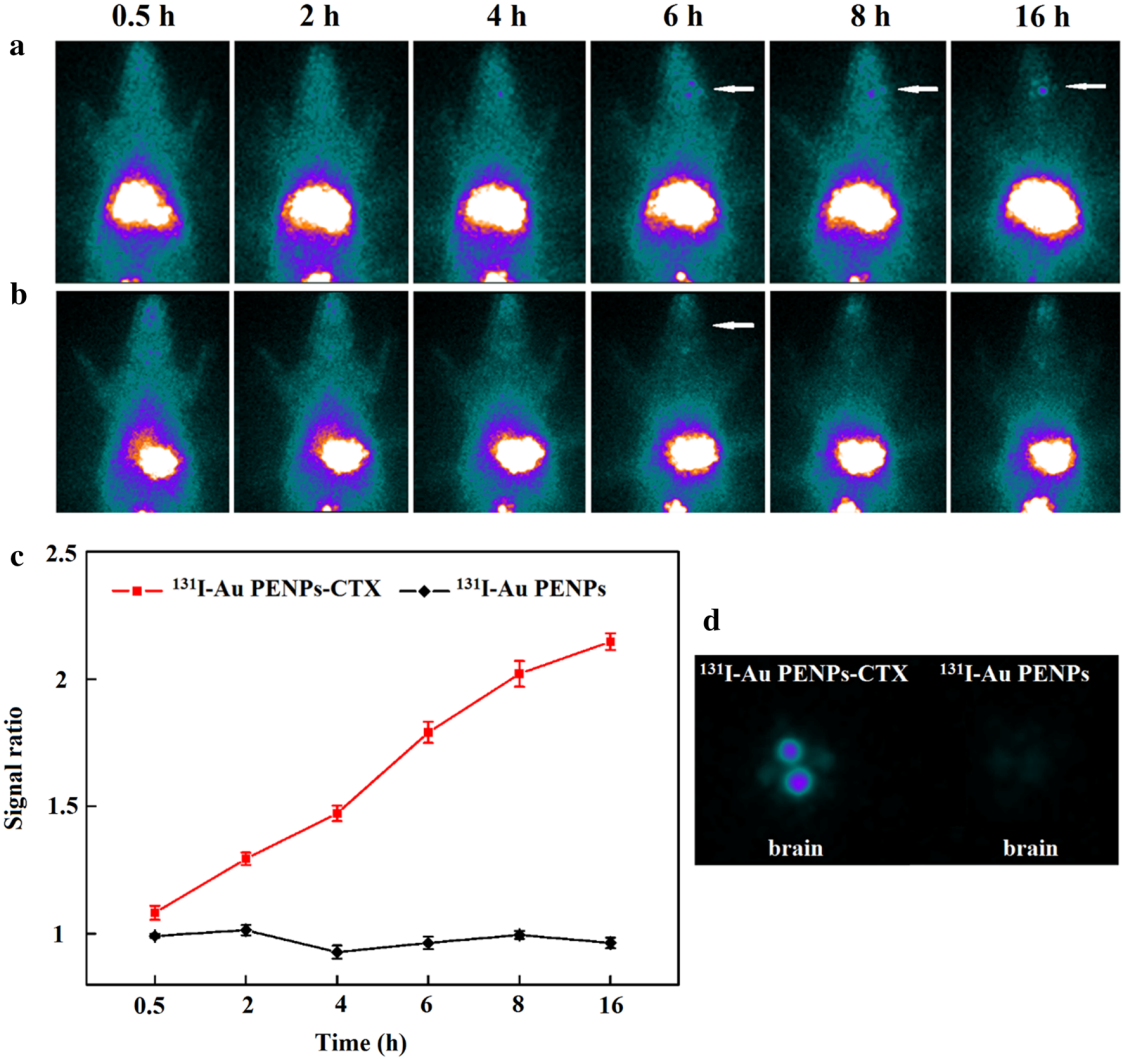
In vivo SPECT images of the rat intracranial glioma model treated with **a** ^131^I-Au PENPs-CTX and **b** ^131^I-Au PENPs at different time points of 0.5, 2, 4, 6, 8 and 16 h, respectively, and **c** their tumor-to-background ratios (TBR) of the SPECT signal intensities in the brains. **d** SPECT images of ex vivo brains at 16 h postinjection

The BBB is the main obstacle to the delivery of nanoparticles into brain tumors. In the early stage, the nanoparticles are able to cross the intact BBB through receptor-mediated endocytosis. In the advanced stage, the BBB becomes defective and the nanoparticles can accumulate in the tumor area via the EPR effect. Given the unique biological properties of CTX, we assessed the targeting ability of CTX-modified Au PENPs to glioma cells in vitro and in vivo in a subcutaneous tumor model. We then evaluated the BBB penetration of ^131^I-Au PENPs-CTX in SD rats bearing intracranial glioma. ^131^I-Au PENPs-CTX and ^131^I-Au PENPs were administrated 14 days after the inoculation of the tumor cells, and their biodistributions in the rats were observed using the same SPECT system. As shown in Fig. 7a, b, at all-time points, the rats injected with ^131^I-Au PENPs-CTX exhibit a higher SPECT signal in the glioma region compared with those injected with ^131^I-Au PENPs without CTX, indicating that CTX modification is able to facilitate the transport of Au PENPs across the BBB and enhance their accumulation at the tumor site. A slightly enhanced tumor uptake can be found at 2 h post-injection, followed by much stronger SPECT signal intensities at 4 and 6 h post-injection. The tumor SPECT signal intensity reaches a peak at 8 h post-injection, and can still be detected at 16 h post-injection. Conversely, no significant levels of radioactivity can be detected in the brains of rats receiving ^131^I-Au PENPs during the period studied. Quantitative SPECT intensity measurements further revealed that the tumor-to-background ratio (TBR) of the SPECT signal in the rats injected with ^131^I-Au PENPs-CTX progressively increased with time (Fig. 7c), while the rats injected with ^131^I-Au PENPs exhibited a consistent TBR because no radioactivity was taken up at the tumor sites. The efficient BBB transport and glioma targeting ability of ^131^I-Au PENPs-CTX were further verified by ex vivo imaging of the brains 16 h post-injection (Fig. 7d). An intense SPECT signal was observed in the brains of rats dosed with ^131^I-Au PENPs-CTX, while little measurable SPECT signal could be detected for rats injected with ^131^I-Au PENPs, suggesting that the rat model possessed an intact BBB and Au PENPs without CTX modification were unable to cross the BBB and target glioma cells even via the EPR effect. These results demonstrate that ^131^I-Au PENPs-CTX could simultaneously achieve BBB transport and glioma targeting.

## Conclusion

We designed and developed PEI-based Au NPs with the ability to traverse the BBB and specifically target brain tumors. PEI surface modified with HPAO and CTX could subsequently be used to entrap Au NPs and then easily labeled with ^131^I via HPAO. Prior to ^131^I radiolabeling, the Au PENPs-CTX exhibited good water dispersibility and stability, favorable X-ray attenuation property and excellent cytocompatibility in the given Au concentration range. The ^131^I-labeled Au PENPs-CTX could be utilized as a nanoprobe for the targeted SPECT/CT imaging and radionuclide therapy of glioma cells in vitro and in a tumor-bearing mouse model in vivo. Furthermore, the developed ^131^I-Au PENPs-CTX were able to cross the BBB and specifically target glioma cells, highlighting their significant potential as a theranostic system for glioma-targeted diagnosis and therapy in vivo. Given that MMP2 is the principal CTX receptor on the surface of tumor cells, the developed CTX-functionalized nanoplatform retains the flexibility to conjugate alternative diagnostic and therapeutic agents for different types of MMP2-overexpressing tumors.

## Additional file

**Additional file 1: Table S1.** The average number of conjugated units on each PEI. **Fig. S1.** ^**1**^H NMR spectra of PEI.NH_2_-(*m*PEG) (a), PEI.NH_2_-(PEG-MAL)-(*m*PEG) (b), PEI.NH_2_-(PEG-CTX)-(*m*PEG) (c), PEI.NH_2_-HPAO-(PEG-CTX)-(*m*PEG) (d), PEI.NH_2_-HPAO-(PEG-MAL)-(*m*PEG) (e), PEI.NH_2_-FI-HPAO-(PEG-CTX)-(*m*PEG) (f), and PEI.NH_2_-FI-HPAO-(PEG-MAL)-(*m*PEG) (g) , respectively. UV-vis spectrum and photograph of Au PENPs-CTX dispersed in water (h). **Fig. S2.** UV-vis spectra of the Au PENPs-CTX dispersed in water at different temperature (a) and pH (b) conditions; Radiochemical purities of the ^131^I-Au PENPs-CTX and ^131^I-Au PENPs exposed to PBS at room temperature (c) and FBS at 37 °C (d) for different time periods. **Fig. S3.** (a) CCK-8 assay of C6 cells treated with the Au PENPs-CTX or Au PENPs at different Au concentrations for 24 h, respectively. (b) CCK-8 assay of C6 cells treated with the ^131^I-Au PENPs-CTX or ^131^I-Au PENPs at different ^131^I concentrations for 24 h, respectively. **Fig. S4.** Flow cytometric analysis of C6 cells incubated with PBS (a), Au PENPs (b) or Au PENPs-CTX (c) at Au concentration of 5 μM for 4 h, respectively. Part (d) shows the comparison of the binding of Au PENPs and Au PENPs-CTX with C6 cells, and the cells treated with PBS were used as controls. **Fig. S5.** Confocal microscopy images of C6 cells treated with PBS, Au PENPs or Au PENPs-CTX with Au concentration of 5 μM for 2 h, respectively. **Fig. S6.** The biodistribution of (a) ^131^I-Au PENPs-CTX and (b) ^131^I-Au PENPs, and (c) their relative signal intensities of different organs at 8 h postinjection. **Fig. S7.** The body weight of C6 tumor-bearing mice after treatments of saline, Au PENPs, Au PENPs-CTX, ^131^I-Au PENPs, and ^131^I-Au PENPs-CTX. Saline was used as control. The relative body weight were normalized according to their initial weights (Mean ± SD, n = 5). **Fig. S8.** Histological changes in the heart, liver, spleen, lung and kidneys of the mice at 2 weeks post-injection of (a) saline, (b) Au PENPs, (c) Au PENPs-CTX, (d) ^131^I-Au PENPs, and (e) ^131^I-Au PENPs-CTX. The organ sections were H&E stained and observed under Leica DM IL LED inverted phase contrast microscope at a magnification of 50 × for each sample (the scale bar in each panel indicates 200 μm).
