# Supplementary material for: Chlorotoxin peptide-functionalized polyethylenimine-entrapped gold nanoparticles for glioma SPECT/CT imaging and radionuclide therapy
Source: J Nanobiotechnology. 2019 Feb 19;17:30. doi: 10.1186/s12951-019-0462-6 (PMC6380014; doi:10.1186/s12951-019-0462-6)
Supplement: Supplementary file 1 — Additional file 1: Table S1. The average number of conjugated units on each PEI. Fig. S1. 1H NMR spectra of PEI.NH2-(mPEG) (a), PEI.NH2-(PEG-MAL)-(mPEG) (b), PEI.NH2-(PEG-CTX)-(mPEG) (c), PEI.NH2-HPAO-(PEG-CTX)-(mPEG) (d), PEI.NH2-HPAO-(PEG-MAL)-(mPEG) (e), PEI.NH2-FI-HPAO-(PEG-CTX)-(mPEG) (f), and PEI.NH2-FI-HPAO-(PEG-MAL)-(mPEG) (g) , respectively. UV-vis spectrum and photograph of Au PENPs-CTX dispersed in water (h). Fig. S2. UV-vis spectra of the Au PENPs-CTX dispersed in water at different temperature (a) and pH (b) conditions; Radiochemical purities of the 131I-Au PENPs-CTX and 131I-Au PENPs exposed to PBS at room temperature (c) and FBS at 37 °C (d) for different time periods. Fig. S3. (a) CCK-8 assay of C6 cells treated with the Au PENPs-CTX or Au PENPs at different Au concentrations for 24 h, respectively. (b) CCK-8 assay of C6 cells treated with the 131I-Au PENPs-CTX or 131I-Au PENPs at different 131I concentrations for 24 h, respectively. Fig. S4. Flow cytometric analysis of C6 cells incubated with PBS (a), Au PENPs (b) or Au PENPs-CTX (c) at Au concentration of 5 μM for 4 h, respectively. Part (d) shows the comparison of the binding of Au PENPs and Au PENPs-CTX with C6 cells, and the cells treated with PBS were used as controls. Fig. S5. Confocal microscopy images of C6 cells treated with PBS, Au PENPs or Au PENPs-CTX with Au concentration of 5 μM for 2 h, respectively. Fig. S6. The biodistribution of (a) 131I-Au PENPs-CTX and (b) 131I-Au PENPs, and (c) their relative signal intensities of different organs at 8 h postinjection. Fig. S7. The body weight of C6 tumor-bearing mice after treatments of saline, Au PENPs, Au PENPs-CTX, 131I-Au PENPs, and 131I-Au PENPs-CTX. Saline was used as control. The relative body weight were normalized according to their initial weights (Mean ± SD, n = 5). Fig. S8. Histological changes in the heart, liver, spleen, lung and kidneys of the mice at 2 weeks post-injection of (a) saline, (b) Au PENPs, (c) Au PENPs-CTX, (d) 131I- [file 12951_2019_462_MOESM1_ESM.docx]

**Additional Information**

**Chlorotoxin peptide-functionalized** **polyethylenimine-entrapped gold nanoparticles for glioma SPECT/CT imaging and radionuclide therapy**

Lingzhou Zhao^1§^, Yujie Li^1§^, Jingyi Zhu^2§^, Na Sun^1^, Ningning Song^1^, Yan Xing^1^, He Huang^2^* and Jinhua Zhao^1^*

^1^ Department of Nuclear Medicine, Shanghai General Hospital, Shanghai Jiao Tong University School of Medicine, Shanghai 200080, People’s Republic of China

^2^ State Key Laboratory of Material-Oriented Chemical Engineering, School of Pharmaceutical Sciences, Nanjing Tech University, Nanjing 211816, People’s Republic of China

________________________________________________________

* Corresponding authors: zhaojinhua1963@126.com (J. Zhao), Fax: 0086-21-37798352, Tel: 0086-21-37798352; huangh@njtech.edu.cn (H. Huang).

^§^These authors equally contributed to this work.

**Part of experimental section**

**Characterization techniques**

^1^H NMR spectra of samples dissolved in D_2_O were obtained using a Bruker AV400 nuclear magnetic resonance spectrometer (Bruker AXS Advanced X-ray Solutions GmbH, Karlsruhe, Germany). UV-vis spectra were measured using a Lambda 25 UV-vis spectrophotometer (PerkinElmer, Inc., Waltham, MA, USA). Dynamic light scattering (DLS) and zeta potential were measured using a Malvern Zetasizer Nano ZS model ZEN 3600 (Malvern Panalytical Ltd., Malvern, UK) with a standard 633 nm laser. The Au content of the prepared PEI-based Au NPs was determined using a Leeman Prodigy inductively coupled plasma optical emission spectrometer (ICP-OES; Teledyne Leeman Labs, Hudson, NH, USA). Transmission electron microscopy (TEM) samples were prepared by dropping an aqueous particle suspension (1 mg/mL) onto a carbon-coated copper grid, followed by air-drying before measurements. TEM imaging was performed using a JEOL 2010F analytical electron microscope (JEOL, Tokyo, Japan) at an operating voltage of 200 kV. The X-ray attenuation properties of the formed Au PENPs-CTX were compared with Omnipaque (iohexol 300; GE Healthcare, Chicago, IL, USA) at different Au or iodine concentrations (6.25-100 μM). CT images were acquired using a GE Discovery STE PET/CT system (GE Healthcare) with 100 kV, 220 mA, and a slice thickness of 1.25 mm. SPECT imaging was performed using a GE Infinia SPECT scanner equipped with an Xeleris workstation and High Energy General Purpose collimators (GE Healthcare).

**Cytotoxicity assay**

C6 glioma cells were cultured in DMEM supplemented with 10% FBS, 100 U/mL penicillin, and 100 μg/mL streptomycin in 5% CO_2_ at 37 ºC. The cytotoxicity of the CTX-modified Au PENPs before and after ^131^I radiolabeling was determined using a CCK-8 assay. Briefly, C6 glioma cells were seeded in 96-well plates (10,000 cells per well) and incubated with 200 μL of DMEM supplemented with 10% FBS overnight. These cells were then treated with ^131^I-Au PENPs-CTX, ^131^I-Au PENPs, Au PENPs-CT, or Au PENPs at different concentrations for 24 h, respectively. After washing with PBS 3 times, CCK-8 solution (100 μL) was added to each well and cultured for 4 h, then the absorbance at 450 nm was measured using a Gen5 microplate reader (BioTek Instruments, Inc., Winooski, VT, USA).

**Confocal microscopy**

Confocal microscopy (Carl Zeiss LSM 700, Jena, Germany) was used to observe the cellular uptake of the Au PENPs-CTX *in vitro*. Briefly, C6 cells were seeded into each well of 12-well plates at a density of 5 × 10^4^ and cultured for 24 h. The medium was replaced with 1 mL of fresh medium containing Au PENPs-CTX (5 μM), Au PENPs (5 μM), or PBS (negative control). After 2 h, the cells were washed with PBS, fixed with 2.5% glutaraldehyde for 15 min at 4 °C, and counterstained with Hoechst 33342 (1 μg/mL) at 37 °C for 20 min. The FI fluorescence was then excited with a 488 nm argon blue laser and a 505-525 nm barrier filter was applied to collect the FI emission. Samples were scanned using a 63× oil-immersion objective lens, and the optical section thickness was set at 5 mm.

**Flow cytometry analysis**

Flow cytometry analysis was carried out to evaluate the targeting efficiency of Au PENPs-CTX towards C6 cells. Briefly, C6 cells were seeded in a 12-well plate (2 × 10^5^ cells per well) and incubated for 24 h. The Au PENPs-CTX or Au PENPs were incubated with the cells to give a final concentration of 5 μM. After 2 h, the cells were harvested and washed 3 times with PBS, and the fluorescence intensities per 10,000 cells were recorded in the FL1-fluorescence channel using a Becton Dickinson FACScan analyzer.

**Table S1**. The average number of conjugated units on each PEI

|  | PEI.NH_2_-FI-HPAO-(PEG-CTX)-(*m*PEG) | PEI.NH_2_-FI-HPAO-(PEG-MAL)-(*m*PEG) |
| --- | --- | --- |
| *m*PEG | 13.3 | 13.3 |
| PEG-MAL | 14.2 | 14.2 |
| CTX | 5.4 | 0 |
| HPAO | 17.8 | 17.3 |
| FI | 3.9 | 3.5 |


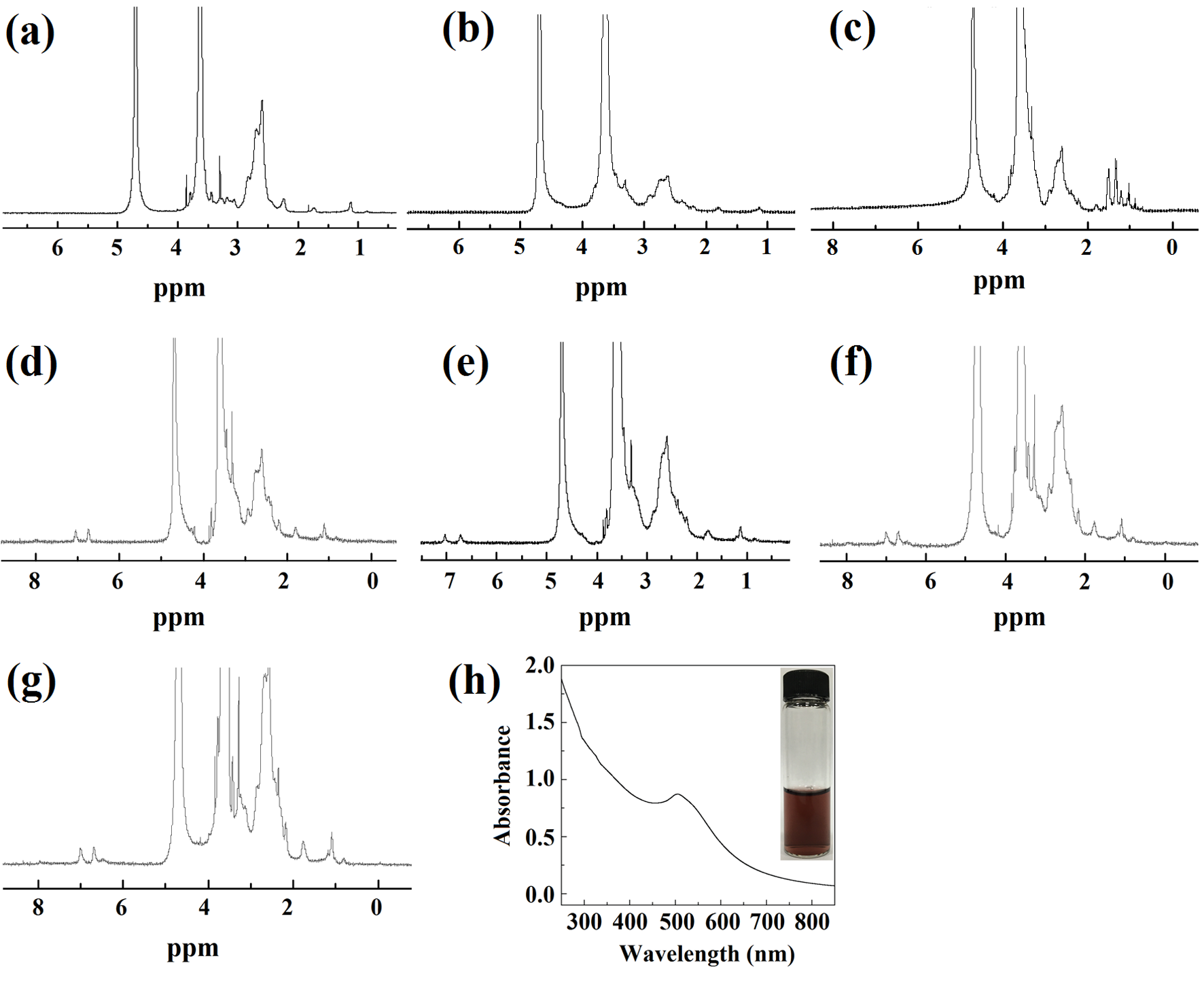


**Fig. S1 ^1^**H NMR spectra of PEI.NH_2_-(*m*PEG) (a), PEI.NH_2_-(PEG-MAL)-(*m*PEG) (b), PEI.NH_2_-(PEG-CTX)-(*m*PEG) (c), PEI.NH_2_-HPAO-(PEG-CTX)-(*m*PEG) (d), PEI.NH_2_-HPAO-(PEG-MAL)-(*m*PEG) (e), PEI.NH_2_-FI-HPAO-(PEG-CTX)-(*m*PEG) (f), and PEI.NH_2_-FI-HPAO-(PEG-MAL)-(*m*PEG) (g) , respectively. UV-vis spectrum and photograph of Au PENPs-CTX dispersed in water (h).


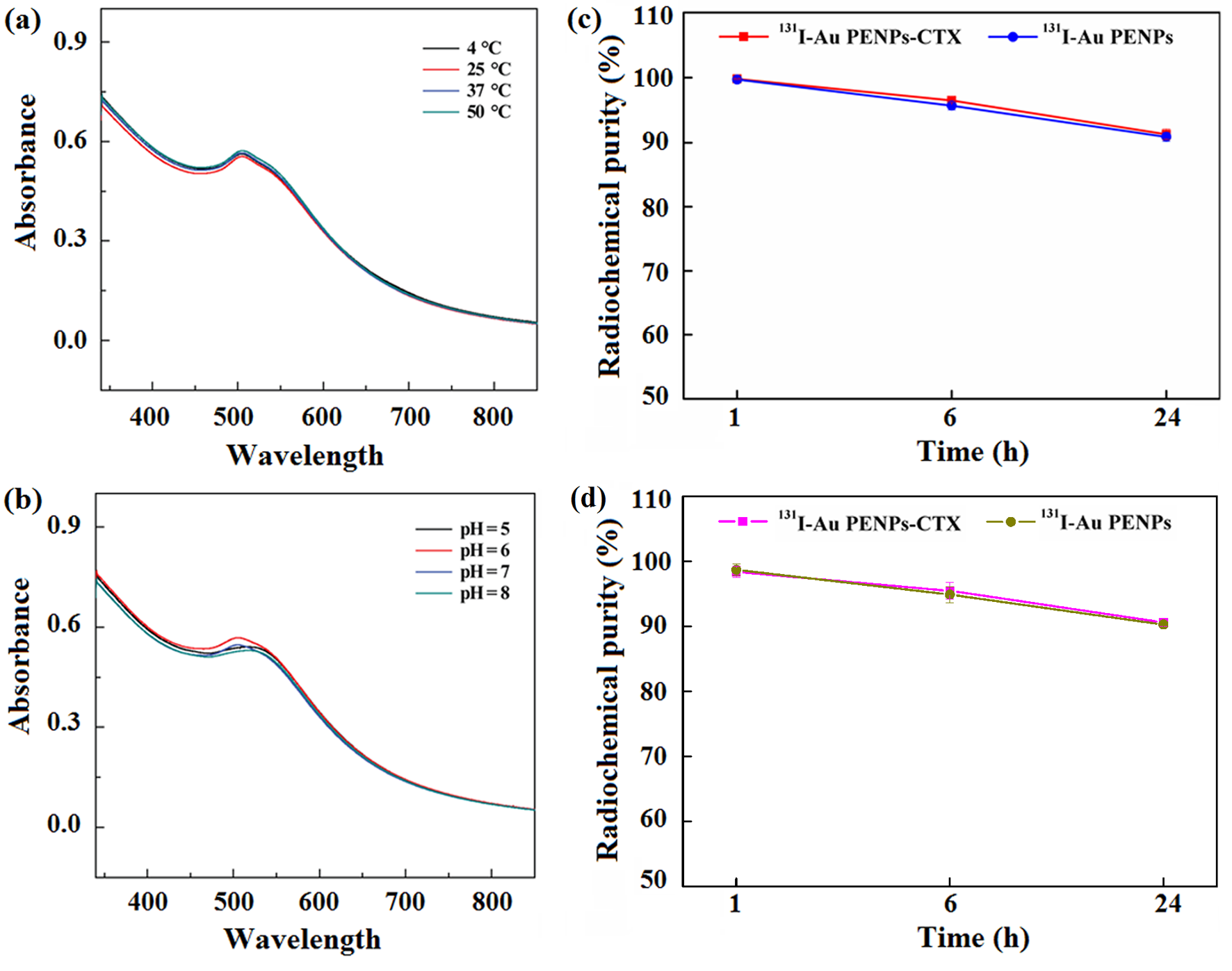


**Fig. S2** UV-vis spectra of the Au PENPs-CTX dispersed in water at different temperature (a) and pH (b) conditions; Radiochemical purities of the ^131^I-Au PENPs-CTX and ^131^I-Au PENPs exposed to PBS at room temperature (c) and FBS at 37 °C (d) for different time periods.


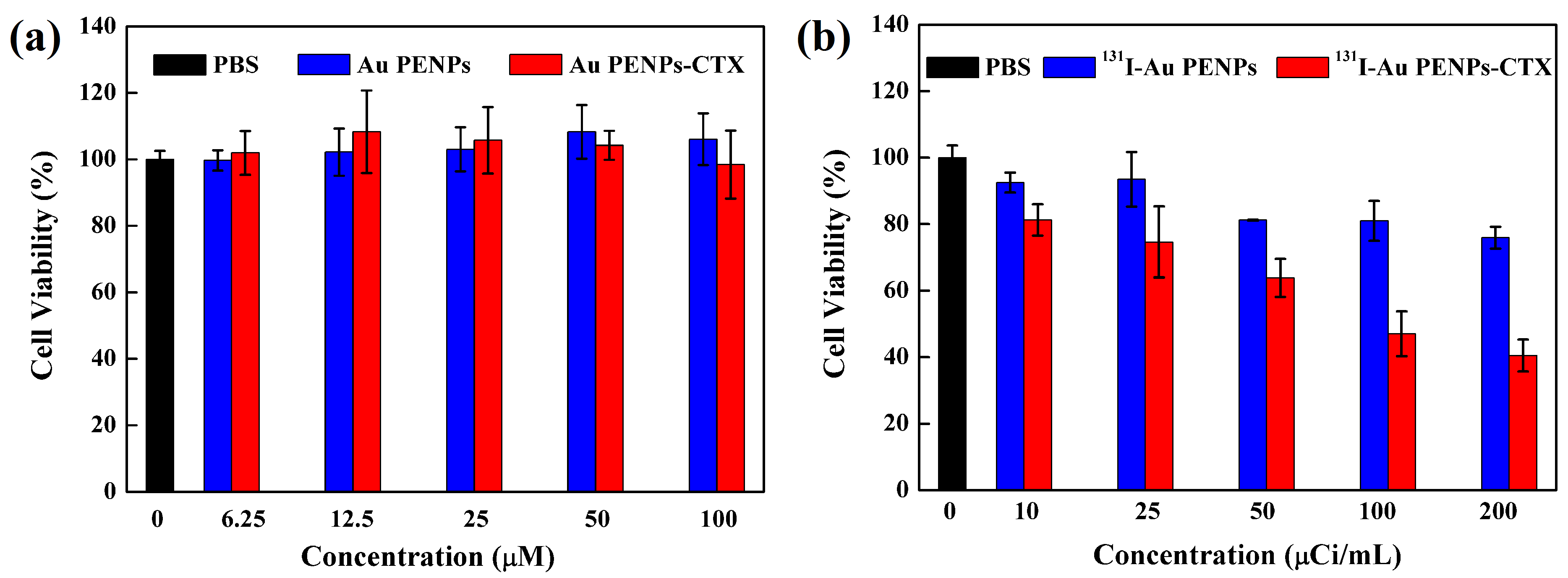


**Fig. S3** (a) CCK-8 assay of C6 cells treated with the Au PENPs-CTX or Au PENPs at different Au concentrations for 24 h, respectively. (b) CCK-8 assay of C6 cells treated with the ^131^I-Au PENPs-CTX or ^131^I-Au PENPs at different ^131^I concentrations for 24 h, respectively.


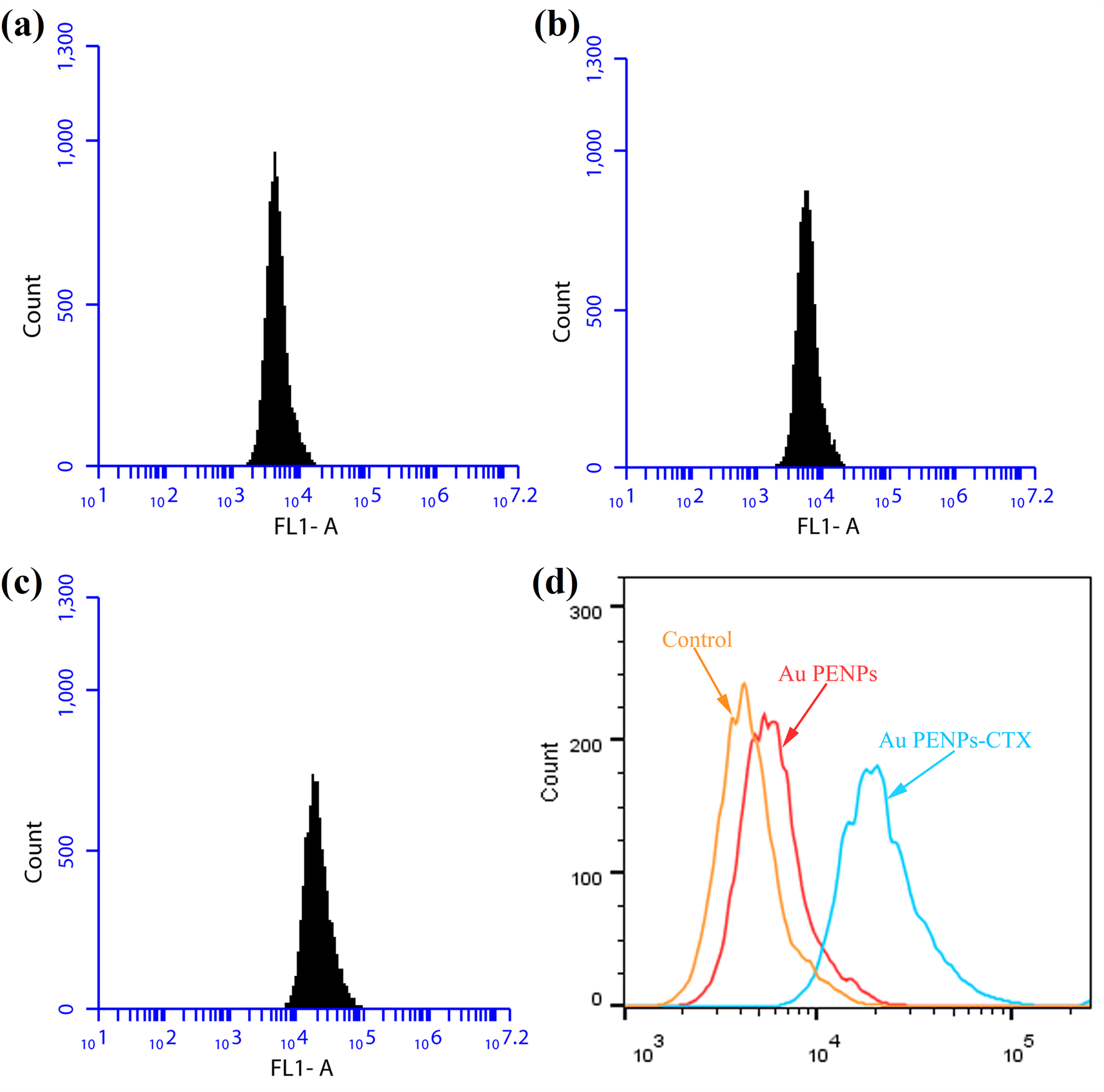


**Fig. S4** Flow cytometric analysis of C6 cells incubated with PBS (a), Au PENPs (b) or Au PENPs-CTX (c) at Au concentration of 5 μM for 4 h, respectively. Part (d) shows the comparison of the binding of Au PENPs and Au PENPs-CTX with C6 cells, and the cells treated with PBS were used as controls.


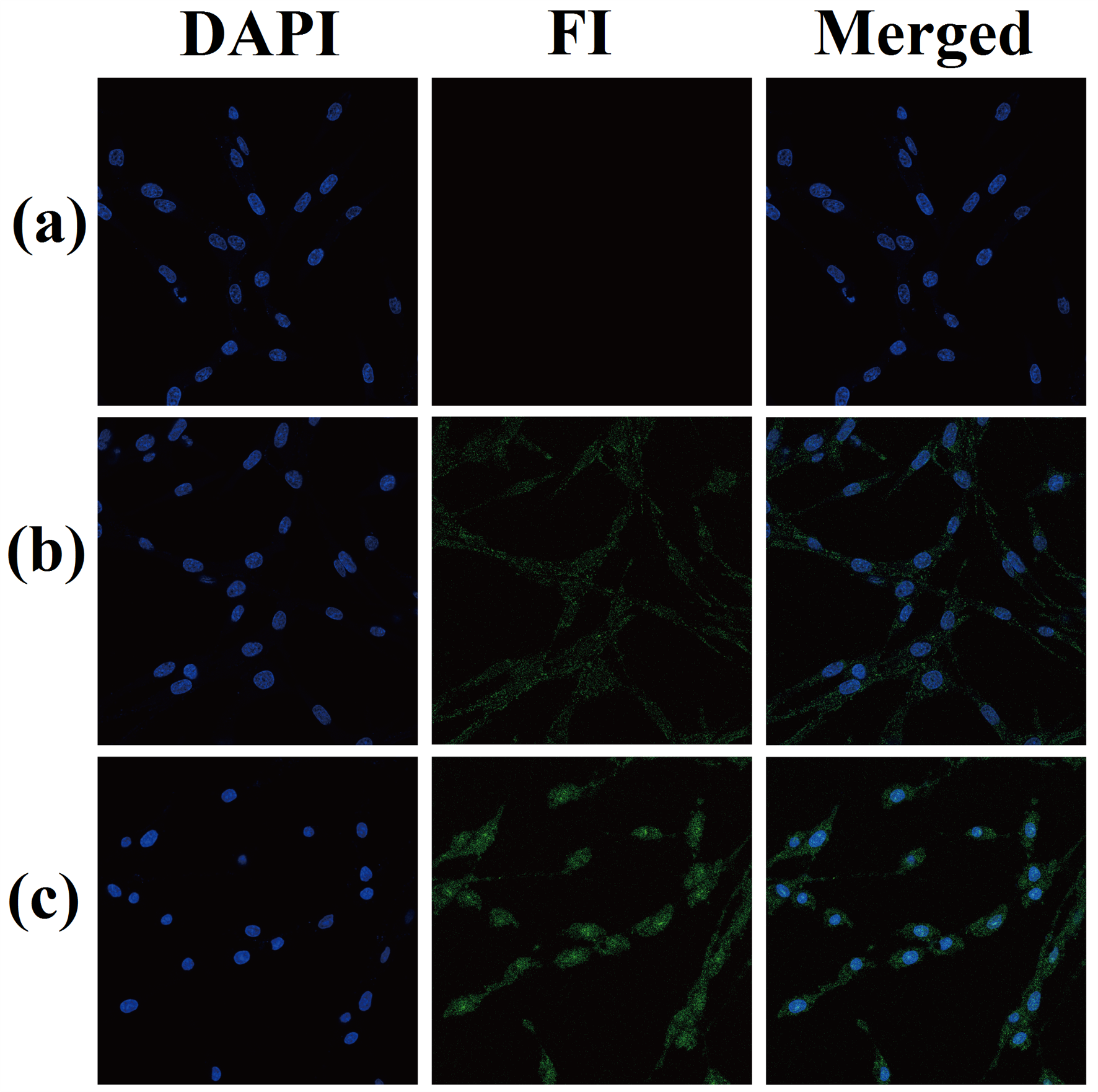


**Fig. S5** Confocal microscopy images of C6 cells treated with PBS, Au PENPs or Au PENPs-CTX with Au concentration of 5 μM for 2 h, respectively.


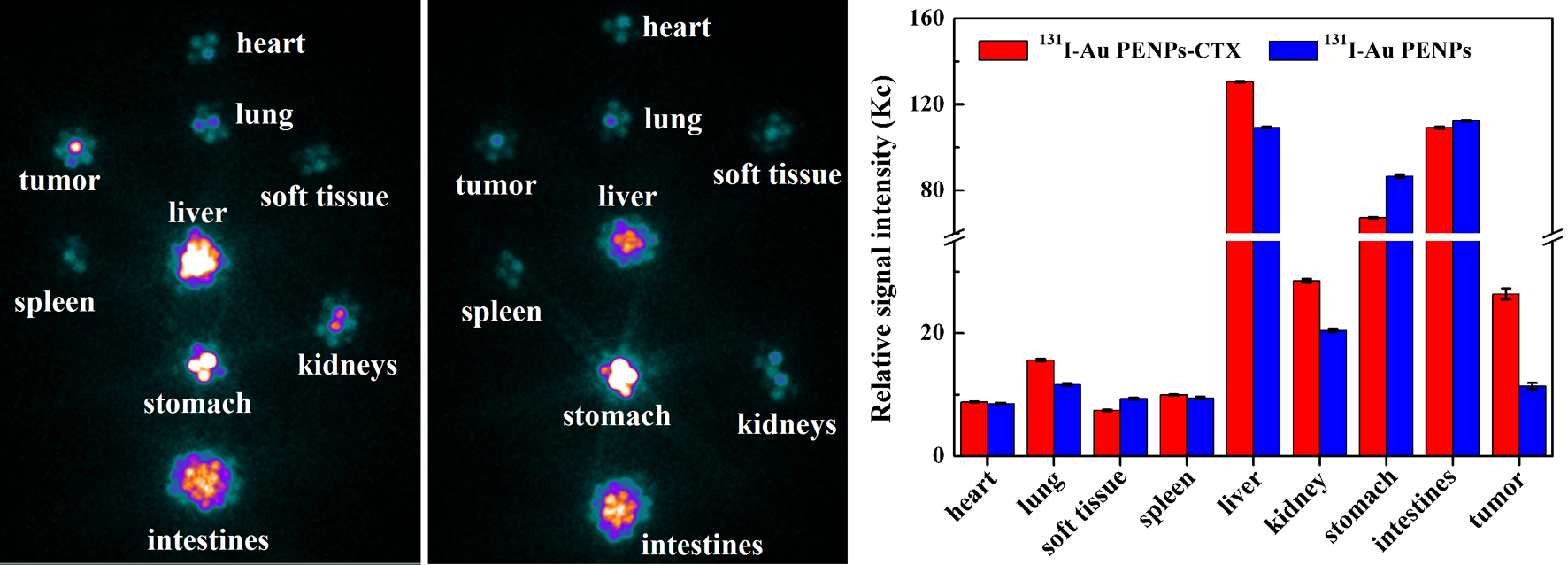


**Fig. S6** The biodistribution of (a) ^131^I-Au PENPs-CTX and (b) ^131^I-Au PENPs, and (c) their relative signal intensities of different organs at 8 h postinjection.


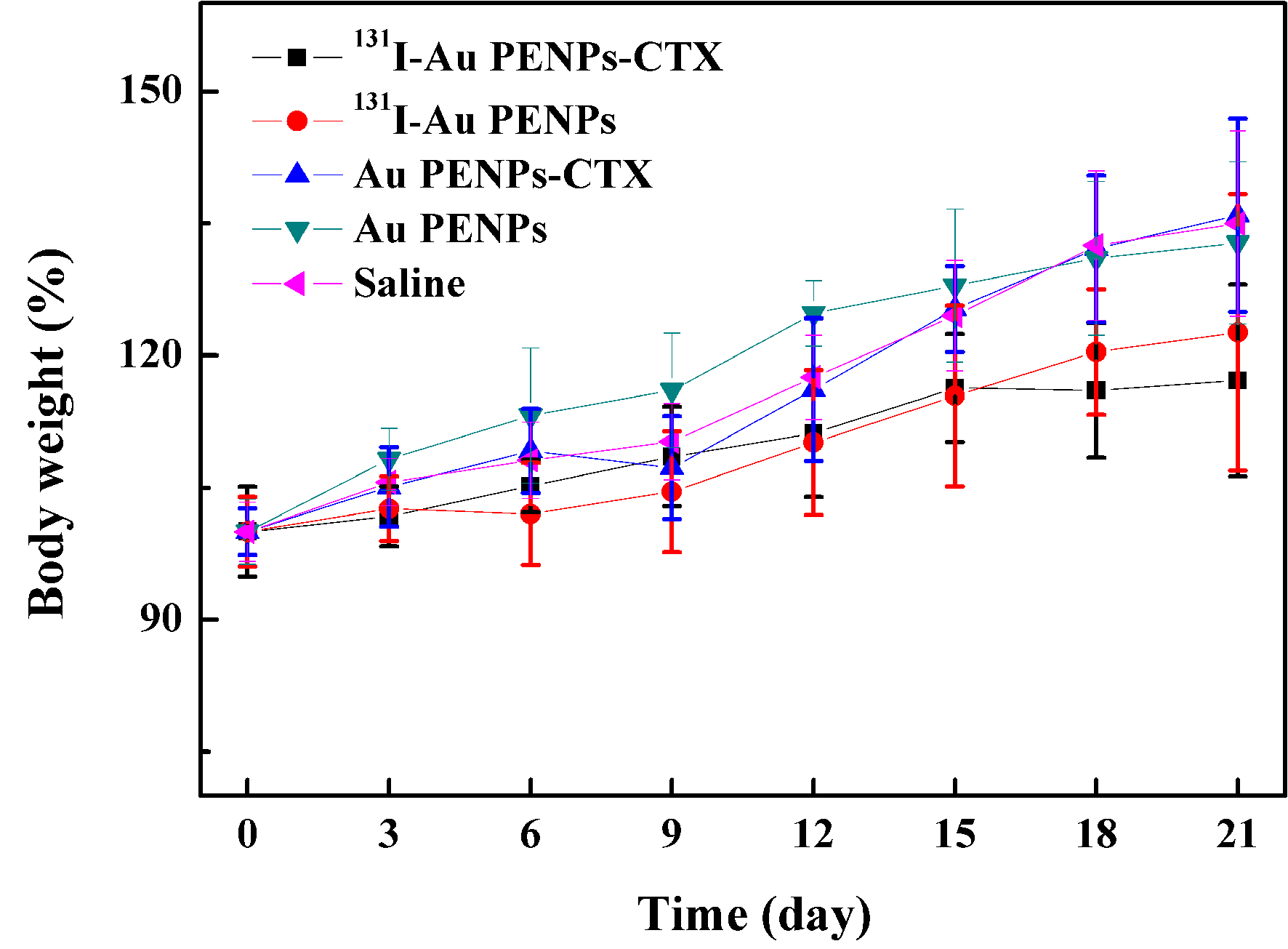


**Fig. S7** The body weight of C6 tumor-bearing mice after treatments of saline, Au PENPs, Au PENPs-CTX, ^131^I-Au PENPs, and ^131^I-Au PENPs-CTX. Saline was used as control. The relative body weight were normalized according to their initial weights (Mean ± SD, n = 5).


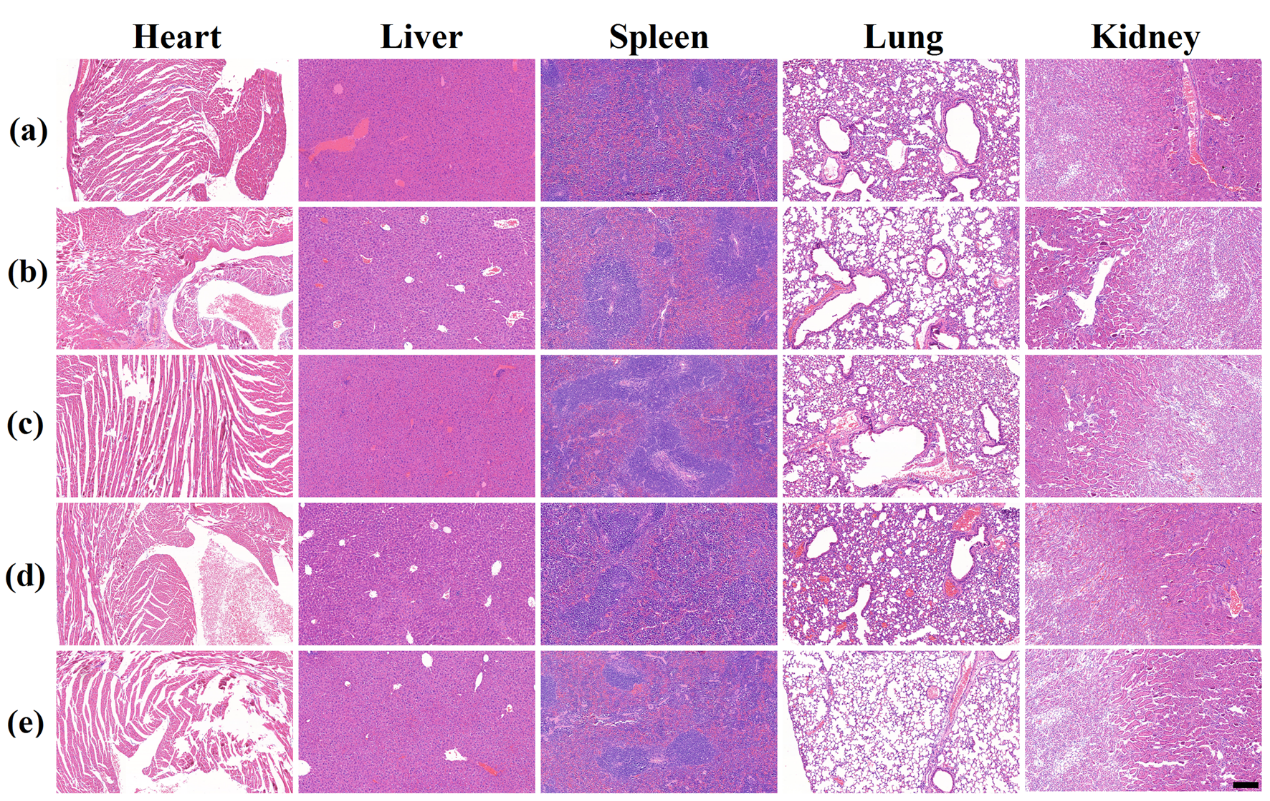


**Fig. S8** Histological changes in the heart, liver, spleen, lung and kidneys of the mice at 2 weeks post-injection of (a) saline, (b) Au PENPs, (c) Au PENPs-CTX, (d) ^131^I-Au PENPs, and (e) ^131^I-Au PENPs-CTX. The organ sections were H&E stained and observed under Leica DM IL LED inverted phase contrast microscope at a magnification of 50 × for each sample (the scale bar in each panel indicates 200 μm).
